# Soluble Transferrin Receptor-1 in Pulmonary Hypertension Associated with COPD

**DOI:** 10.1007/s00408-025-00833-3

**Published:** 2025-07-14

**Authors:** Oleh Myronenko, Pero Curcic, Philipp Douschan, Katarina Zeder, Teresa John, Susanne Suessner, Konrad Hoetzenecker, Gabor Kovacs, Andrea Olschewski, Horst Olschewski, Vasile Foris

**Affiliations:** 1 Division of Respiratory Medicine, Lung Research Cluster, Department of Internal Medicine, Medical University of Graz, Auenbruggerplatz 15, 8036 Graz, Austria; 2 European Research Network Lung Affiliated Pulmonary Hypertension National Expert Center, Graz, Austria; 3 Clinical Institute of Medical and Chemical Laboratory Diagnostics, Medical University of Graz, Graz, Austria; 4 Institute for Health Computing, University of Maryland, Bethesda, MD USA; 5 Blutzentrale Linz, Austrian Red Cross, Linz, Austria; 6 Department of Thoracic Surgery, Medical University of Vienna, Vienna, Austria; 7 Department of Anaesthesiology and Intensive Care Medicine, Medical University of Graz, Graz, Austria; 8 Sigmund Freud Private University Vienna, Vienna, Austria; 9 Charité University Medicine, Berlin, Germany; 10 Channing Division of Network Medicine, Department of Medicine, Brigham and Women´S Hospital, Harvard Medical School, Boston, MA USA

**Keywords:** Biomarker, Pulmonary hypertension, COPD, Iron, Soluble transferrin receptor-1

## Abstract

**Purpose:**

The pulmonary vascular involvement of COPD ranges from severe airway obstruction without pulmonary hypertension (PH) to mild airway obstruction with severe PH. Iron-dependent molecular regulators like hypoxia-inducible factor-2 (HIF-2) may contribute to these phenotypic variations. We explored the role of soluble transferrin receptor-1 (sTfR1) for diagnosis and prognosis of PH associated with COPD.

**Methods:**

We analyzed COPD outpatients who underwent right heart catheterization and performed unsupervised clustering analysis based on sTfR1 levels and non-invasive clinical parameters to identify specific COPD phenotypes. Additionally, we examined explanted end-stage COPD lungs for TfR1 expression and iron deposition.

**Results:**

sTfR1 was associated with mean pulmonary artery pressure (mPAP) (*r* = 0.45, *p* < 0.001), pulmonary vascular resistance (PVR) (*r* = 0.395, *p* < 0.001) and poor survival. sTfR1 predicted severe PH (AUROC [95% CI], 0.72 [0.61–0.83]), and AUROC was further improved based on a combination of sTfR1 and low hemoglobin (0.82 [0.73–0.92]). sTfR1-based cluster analysis distinguished three COPD phenotypes with significantly different survival. One of the two clusters with poor survival was characterized by moderate airway obstruction and moderate PH but elevated sTfR1, anemia and inflammation. In explanted COPD lungs, TfR1-positive and iron-laden cells, most likely, consisted of macrophages, and iron-loaded cell density was negatively correlated with mPAP in patients with moderate/severe PH (*r* = − 0.681, *p* = 0.015).

**Conclusions:**

Elevated sTfR1 predicts poor prognosis in patients with COPD and, particularly in combination with low hemoglobin, may serve as a biomarker for severe PH in COPD, identifying a distinct phenotype with systemic inflammation, anemia and iron deficiency.

**Supplementary Information:**

The online version contains supplementary material available at 10.1007/s00408-025-00833-3.

## Introduction

Up to 39% of patients with chronic obstructive disease (COPD) present with pulmonary hypertension (PH), a combination that is associated with poor prognosis [1–4]. However, PH associated with COPD (COPD-PH) contains various phenotypes [5, 6], and only a small subset of patients is characterized by severe PH with pulmonary vascular resistance (PVR) > 5 WU [7], which may represent an indication for individualized management in PH expert centers including consideration of targeted pulmonary arterial hypertension (PAH) therapy [8]. The diagnosis of severe PH requires right heart catheterization (RHC), highlighting the need for reliable non-invasive blood-derived diagnostic markers.

The mechanisms underlying severe PH in COPD remain poorly understood. In the mouse model, upregulation of the iron-dependent hypoxia-inducible factor-2 (HIF-2) in pulmonary arterial endothelial cells is associated with more pronounced vascular remodeling and severe PH [9], whereas its knockout causes emphysema [10]. In turn, HIF-2 activity is tightly regulated by iron at both post-transcriptional and post-translational levels [11]. Notably, up to 50% of COPD patients exhibit either anemic or non-anemic iron deficiency [12], which is linked to more severe PH [13, 14]. Conversely, COPD lungs contain higher iron levels compared to healthy controls, with predominant deposition in the bronchial epithelium and alveolar macrophages [15]. However, the mechanisms driving iron accumulation in the lung, the involvement of the pulmonary vasculature in this process and the contribution of iron-related factors to COPD phenotypes remain elusive. Therefore, dysregulated iron homeostasis in COPD, both systemically and within the lungs, may contribute to disease pathogenesis, making iron-dependent molecular factors attractive targets for biomarker discovery.

The Pulmonary Vascular Disease Phenomics (PVDOMICS) study demonstrated that iron deficiency was correlated with reduced exercise capacity, right ventricular remodeling and poor survival [16]. However, soluble transferrin receptor-1 (sTfR1), a key indicator of systemic iron demand [17], was not assessed. Recently, elevated sTfR1 has been linked to increased all-cause mortality in chronic kidney disease [18]. Interestingly, in a large plasma proteome study, sTfR1 was associated with early COPD onset and smoking [19].

We assessed sTfR1 in a real-world cohort of COPD outpatients enriched with severe PH to determine its association with pulmonary hemodynamics and prognosis. Additionally, we investigated iron status in explanted lung tissue of end-stage COPD patients by means of transferrin receptor-1 (TfR1/CD71) expression and iron deposition.

## Methods

### Outpatient Cohort

Serum samples were collected from COPD patients who underwent RHC at our PH Clinic in Graz, Austria between 2011 and 2019. The inclusion criteria for the COPD-PH groups were in accordance with the updated definition of PH in the 2022 ESC/ERS Guidelines for the diagnosis and treatment of PH [8]. Subjects were primarily categorized into three groups based on the magnitude of pulmonary pressure elevation (mean pulmonary artery pressure, mPAP): No PH with mPAP ≤ 20 mmHg, mild-moderate PH with mPAP = 21–34 mmHg and severe PH with mPAP ≥ 35 mmHg. In a secondary analysis, patients were grouped according to their pulmonary vascular resistance (PVR) (PVR ≥ 5 WU vs. PVR < 5 WU). All patients were free of intercurrent infection and in clinically stable state at the time of RHC. The protocol and use of the samples were approved by the institutional ethics committee (23–408 ex 10/11). All participants signed written informed consent forms before inclusion. Freshly obtained serum samples were aliquoted and stored in our Biobank at − 80 °C until further processing.

### Lung Transplant Cohort (LTX)

Lung tissue samples were obtained from end-stage COPD patients who underwent RHC in the framework of lung transplantation between 2011 and 2020. Owing to the lack of reliable PVR data for the whole cohort and the limited number of COPD patients without PH, patients were categorized into three groups based on mPAP at rest: No or mild PH with mPAP < 25 mmHg, moderate PH with mPAP = 25–34 mmHg and severe PH with mPAP  ≥ 35 mmHg. Exclusion criteria were a diagnosis of other respiratory diseases (cystic fibrosis, sarcoidosis, tuberculosis, etc.) and/or severe systemic diseases (e.g., scleroderma). The protocol and use of the samples were approved by the institutional ethics committee of the transplant center (976/2010), and written informed consent was obtained from all patients prior to enrollment. After processing, the serum samples were stored at − 80 °C, and the lung tissue samples were fixed in 4% paraformaldehyde and embedded in paraffin until further use, as previously described [20].

#### Biomarker Assessment

Soluble transferrin receptor-1 (sTfR1) was measured on the Cobas 8000 System analyser (Roche Diagnostics, Germany) using the particle-enhanced immunoturbidimetric assay kit Tina-quant® Soluble Transferrin Receptor-1 ver.2 (sTFR2, cat. #07227892190, Roche Diagnostics, Germany). Briefly, latex-bound antibodies react with the antigen in the sample forming antigen–antibody complexes that agglutinate and change the turbidity of the medium, which is measured photometrically. Hemoglobin, hematocrit, red blood cell (RBC) count, red cell distribution width (RDW), C-reactive protein (CRP) and NT-proBNP were measured as a part of routine clinical assessments on the day of RHC.

#### Lung Tissue Staining

Immunohistochemical (IHC) staining for transferrin receptor-1 (CD71) was conducted on formalin-fixed paraffin-embedded tissue sections using a polyclonal goat antibody against CD71 at 1:200 dilution (cat. #AF2474, RRID: AB_416601, R&D Systems, USA), followed by a biotinylated donkey anti-goat secondary IgG antibody (cat. #BA–9500–1.5, Vector Laboratories, USA) and an avidin–biotin complex HRP detection system from the ImmPACT NovaRED® Substrate Kit (cat. #SK-4805, Vector Laboratories, USA), as previously described [21]. An Iron Stain Kit (cat. #ab150674, Abcam, UK) was used for ferric iron detection in lung tissue utilizing Perls´ Prussian blue method with pararosaniline counterstaining following the manufacturer’s instructions. The number of positive cells represents the mean of CD71-positive cells or iron-loaded cells per 1 mm^2^ from five different areas, randomly selected on each lung section. Two random sections from two random blocks from each lung were stained and quantified. The positive pixel densities of the scanned slides were analyzed using QuPath v.0.4.3 (open-source software) [22].

#### Statistical and Bioinformatics Analyses

Data distribution was assessed using the one-sample Kolmogorov–Smirnov test. Continuous variables are presented as medians with interquartile ranges (IQR, Q1-Q3). Group comparisons were conducted using the Mann–Whitney U test (for two independent groups) and the Kruskal–Wallis H test with Dunn´s post hoc test (for multiple comparisons). Categorical variables were compared using the Chi-square test. Correlations were analyzed with Spearman´s rank test. A Generalized Additive Model (GAM) was applied to evaluate the non-linear relationship between mPAP and sTfR1 levels. Biomarker performance was tested by calculating the area under the receiver operating characteristic curve (AUROC). Comparisons of AUROC were performed using DeLong´s test. Survival, defined from the date of the initial RHC (which corresponds to the date of blood drawing) to death (all-cause mortality) or lung transplantation, was analyzed using Kaplan–Meier curves and multivariate Cox regression, adjusted for age and FEV1. Vital status was available until December 31, 2023, as provided by Statistics Austria. Principal component analysis (PCA) was performed using the ‘*prcomp*’ function in R, incorporating age and the available non-invasive clinical parameters (Table 1). Significant PCA vectors were selected based on a loading threshold of 0.5 to highlight the variables with substantial contributions to the principal components. Standardized PCA-transformed data were clustered using the k-means algorithm, with the optimal number of clusters determined by the silhouette method (Rousseeuw, 1987). Statistical analysis was performed using IBM SPSS Statistics v.29.0.0 (IBM SPSS Inc., USA) and R v.4.4.1 (R Foundation for Statistical Computing, Vienna, Austria). *P*-values < 0.05 were considered statistically significant.

**Table 1:** Demographic and clinical characteristics of the outpatient cohort

	COPD (no PH)	COPD-PH mild/moderate	COPD-PH severe	*P-value*
*N*	10	28	41	*NA*
Sex (*n*), M:F	4:6	13:15	28:13	*0.101*
Age, year	65.0 (61.3—76.5)	70.0 (65.0—73.0)	68.0 (62.0—73.0)	*0.524*
BMI, kg/m^2^	22.2 (19.2—28.0)	24.7 (20.5—28.7)	27.9 (22.8—31.9)	*0.022*
mPAP, mmHg	18.0 (15.5—19.0)	26.0 (24.0—29.0)	43.0 (36.5—51.5)	< *0.001*
PVR, Wood unit	2.2 (1.6—3.2)	3.9 (2.9—4.6)	7.8 (5.2—10.2)	< *0.001*
PAWP, mmHg	8.0 (5.8—9.3)	9.0 (7.0—11.0)	10.0 (8.5—12.5)	*0.012*
CI, L/min/m^2^	2.6 (2.4—2.9)	2.6 (2.3—3.0)	2.3 (1.9—2.7)	*0.102*
FEV1, % pred	79.2 (38.3—88.2)	61.1 (38.3—69.6)	52.6 (39.3—65.8)	*0.250*
FEV1/FVC	0.58 (0.33—0.66)	0.60 (0.46—0.64)	0.55 (0.47—0.64)	*0.946*
GOLD stage (n), 1:2:3:4	5:2:1:2	4:15:6:3	5:19:13:4	*0.085*
DLCO, % pred	63.6 (36.7—88.7)	47.7 (33.4—78.0)	38.7 (22.9—58.7)	*0.031*
RBC, million/mm^3^	4.5 (4.3—4.6)	4.5 (4.1—4.7)	4.7 (4.3—5.0)	*0.177*
Hematocrit, %	41.2 (39.0—42.4)	39.8 (37.2—42.4)	43.0 (37.4—47.6)	*0.052*
Hemoglobin, g/dL	13.5 (12.8—14.3)	13.1 (12.2—14.4)	14.6 (12.8—15.7)	*0.064*
RDW, %	13.5 (12.5—14.1)	14.4 (13.7—15.4)	15.2 (14.3—16.9)	< *0.001*
NT-proBNP, pg/mL	101 (49.8—522.3)	222 (108.3—638.5)	1516 (418.5—2641.5)	< *0.001*
CRP, mg/L	1.2 (0.7—3.7)	2.4 (1.1—5.9)	5.5 (1.8—11.6)	*0.008*

## Results

The demographic and clinical characteristics of the outpatient cohort (*n* = 79) are summarized in Table 1.

Data are presented as n or median with interquartile ranges (IQR, Q1-Q3). Differences between groups are assessed using the Chi-square and the Kruskal–Wallis tests. *BMI* body mass index, *mPAP* mean pulmonary artery pressure, *PVR* pulmonary vascular resistance, *CI* cardiac index, *FEV1* forced expiratory volume in 1 s, *FEV1/FVC* forced expiratory volume in 1 s to forced vital capacity ratio, *GOLD* Global Initiative for Chronic Obstructive Lung Disease, *DLCO* diffusing capacity of the lung for carbon monoxide, *RBC* red blood cell count, *RDW* red blood cell distribution width, *NT-proBNP* N-terminal pro B-type natriuretic peptide, *CRP* C-reactive protein, *NA* not applicable, *pred* predicted

### Circulating Soluble Transferrin Receptor-1 in COPD Outpatients

**Fig. 1:**
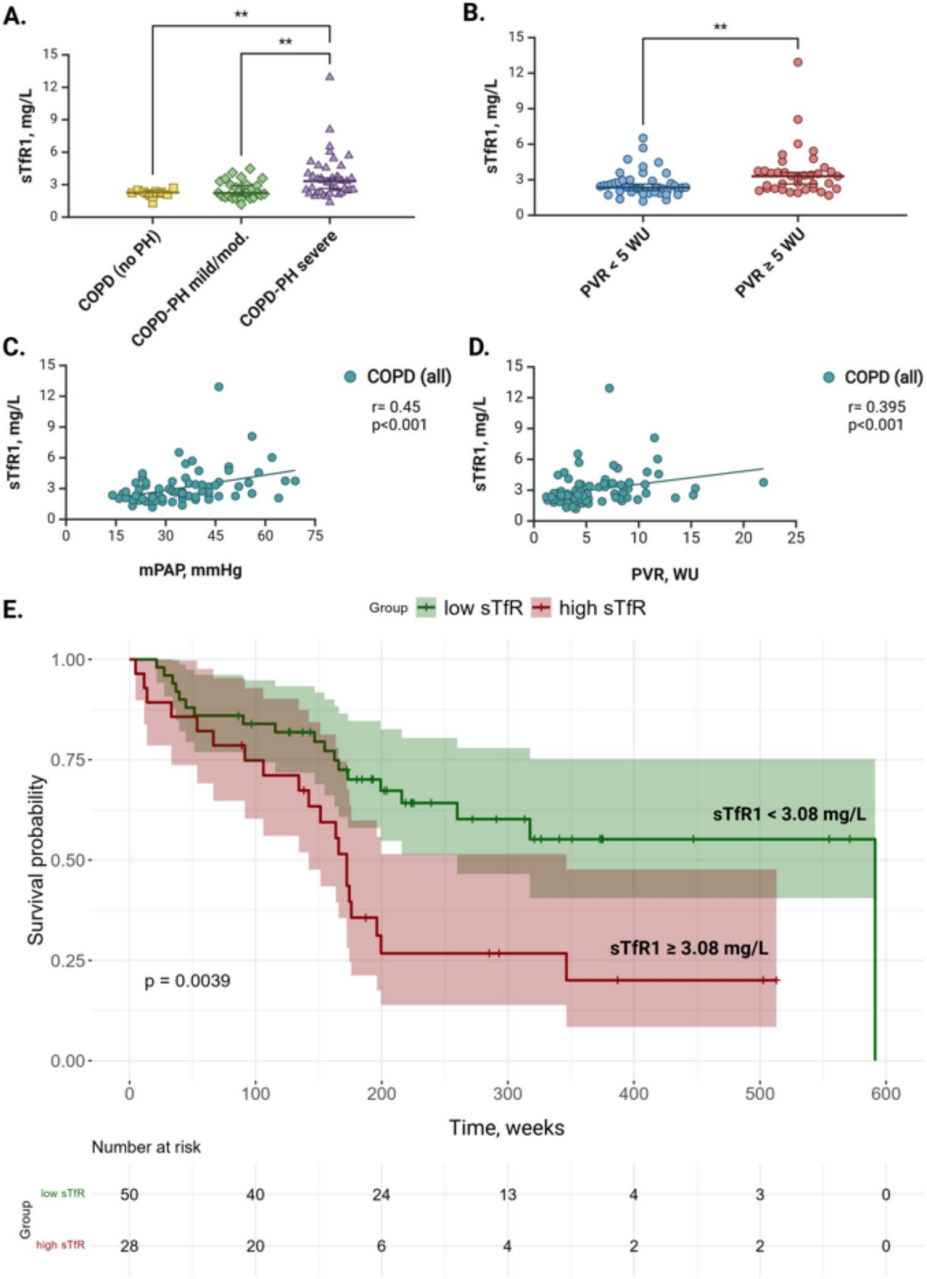
Soluble transferrin receptor-1 in COPD outpatients. (**A**) Comparison of sTfR1 levels between COPD subgroups. (**B)** Comparison of sTfR1 levels between COPD patients with low and high pulmonary vascular resistance (PVR < 5 vs. ≥ 5 WU, respectively). (**C**) Correlation between sTfR1 and mPAP. (**D**) Correlation between sTfR1 and PVR. (**E**) Kaplan–Meier survival curves grouped by mean sTfR1 (sTfR1 ≥ 3.08 mg/L vs. sTfR1 < 3.08 mg/L), with 95% CI and time in weeks. WU, Wood unit; mPAP, mean pulmonary artery pressure; sTfR1, soluble transferrin receptor-1. *P < 0.05, **P < *0.01*

COPD outpatients with severe PH had elevated sTfR1 levels (3.32 mg/L, IQR: 2.36–4.30) compared to those with mild/moderate PH (2.23 mg/L, IQR: 1.87–2.98; *p* = 0.005) and no PH (2.27 mg/L, IQR: 1.98–2.39; *p* = 0.008) (Fig. 1A). Accordingly, sTfR1 levels were increased in patients with PVR ≥ 5 WU compared to PVR < 5 WU (3.3 mg/L, IQR: 2.26–3.75 *vs.* 2.35 mg/L, IQR: 1.96–2.92; *p* = 0.004) (Fig. 1B), and correlated with mPAP (*r* = 0.45; *p* < 0.001), PVR (*r* = 0.395; *p* < 0.001) (Fig. 1C, D), NT-proBNP (*r* = 0.419; *p* < 0.001) and cardiac index (*r* = − 0.255; *p* = 0.023). Generalized Additive Model (GAM) analysis confirmed a non-linear relationship between sTfR1 and mPAP (F = 7.62, *p* < 0.001) (e-Fig. 1A–E).

Using the sTfR1 mean value (3.08 mg/L) as a cut-off, we performed survival analysis. The median follow-up time was 3.5 years. Increased sTfR1 levels were associated with poorer survival (*p* = 0.0039), independent of age and FEV1 (Fig. 1E).

**Fig. 2:**
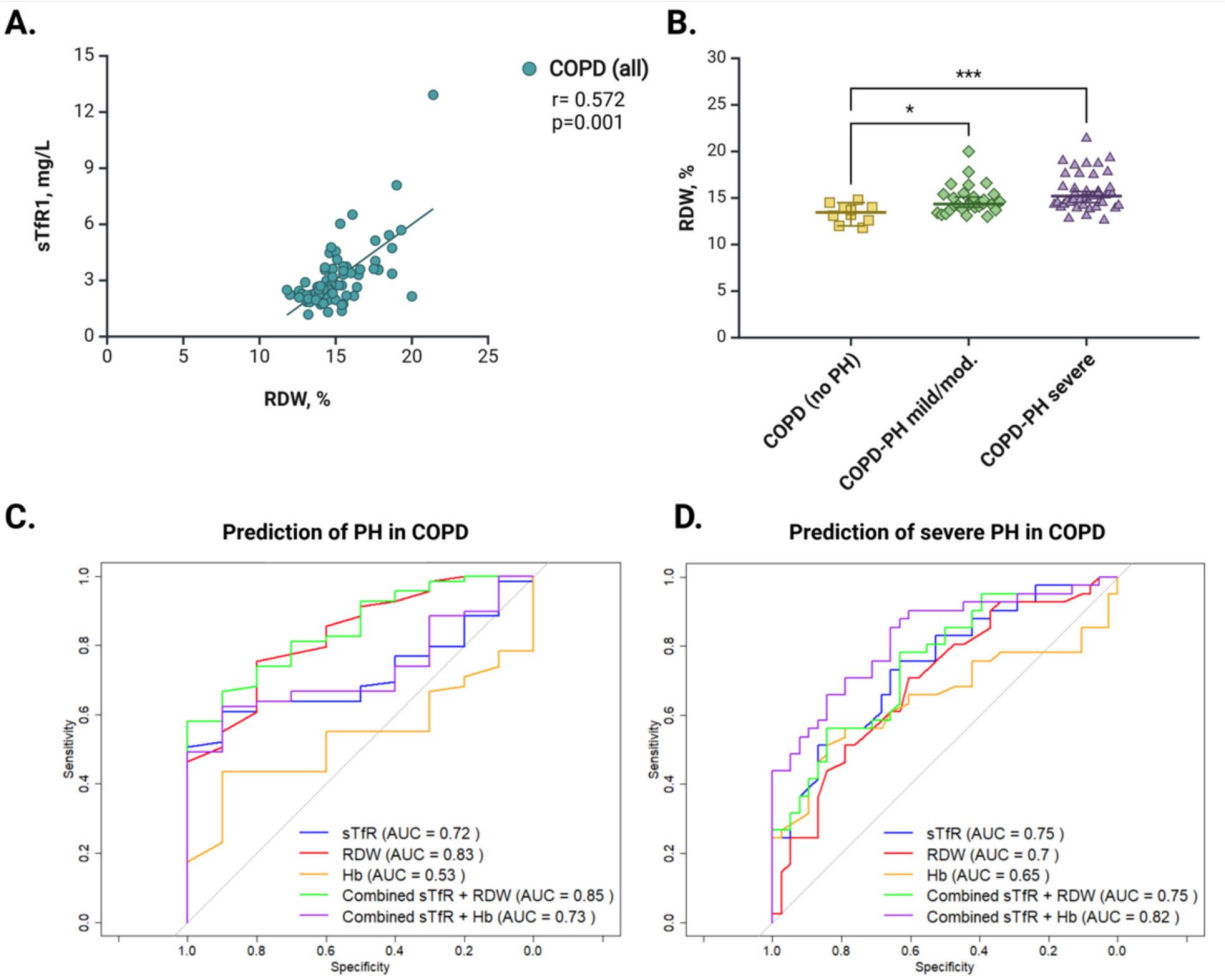
sTfR1 and RDW in COPD subgroups. (**A**) Correlation between sTfR1 levels and RDW in all COPD patients. (**B**) RDW levels in COPD subgroups of the outpatient cohort. (**C**) Receiver operating characteristic (ROC) curves showing the predictive value of sTfR1, RDW, hemoglobin and their combination for PH. (**D**) Receiver operating characteristic (ROC) curves showing the performance of sTfR1, RDW, Hb and their combinations as predictors of severe PH. Hb, hemoglobin; RDW, red blood cell distribution width; mPAP, mean pulmonary artery pressure. *P < 0.05, **P < 0.01, ***P < *0.001*

In addition, sTfR1 levels were positively correlated with RDW (*r* = 0.572; *p* = 0.001) (Fig. 2A) but not with hemoglobin, hematocrit or RBC count. Unlike sTfR1, RDW was different only between the no PH and the severe PH group (*p* = 0.001) (Fig. 2B). The predictive values for PH of sTfR1, RDW and their combination were similar (*p* > 0.05): The areas under the receiver operating characteristic curves (AUROC [95% CI]) were 0.72 [0.61–0.83], 0.83 [0.71–0.95] and 0.85 [0.74–0.96], respectively (Fig. 2C). For the prediction of severe PH, the combination of high sTfR1 with low hemoglobin increased AUROC (0.82 [0.73–0.92]) compared to sTfR1 (0.75 [0.64–0.85]), RDW (0.70 [0.58–0.81]), hemoglobin (0.65 [0.52–0.77]) alone, and to the combination of sTfR1 with RDW (0.75 [0.64–0.86]) (Fig. 2D). Subgroup analyses for patients with and without anemia as well as underweight, non-obese and obese patients are presented in the Supplement (e-Fig. 2).

### Cluster Analysis of COPD Outpatients

**Fig. 3:**
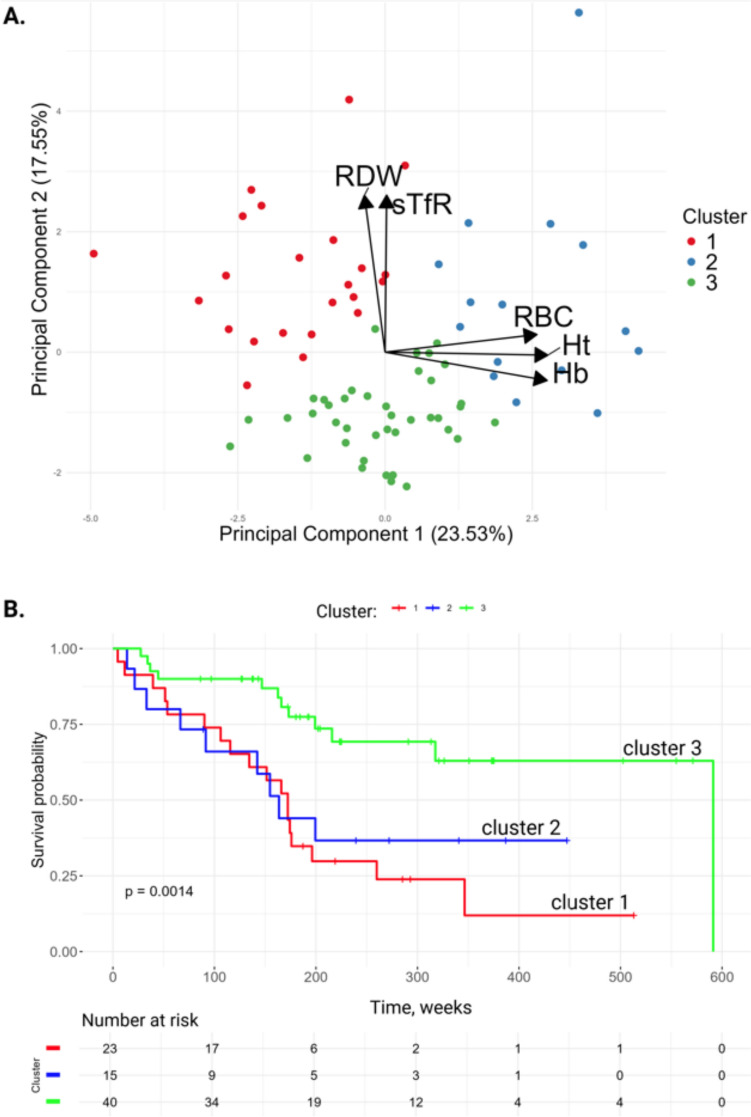
Principal component analysis and unsupervised clustering. (**A**) Principal Component Analysis (PCA) and unsupervised clustering analysis of the COPD outpatient cohort, with clusters color-coded and significant vectors indicating the key clinical parameters contributing to the differences. (**B**) Kaplan–Meier survival curves for different clusters of the outpatient cohort with the total log-rank *p*-value. *RDW* red blood cell distribution width, *sTfR1* soluble transferrin receptor-1, *RBC* red blood cell, *Ht* hematocrit, *Hb* hemoglobin, *FEV1* forced expiratory volume in 1 s

To reduce dimensionality of the complex interactions between clinical parameters and to identify key non-invasive variables that could differentiate clinically relevant COPD subgroups, we performed principal component analysis (PCA) and unsupervised clustering based on the sTfR1 values and the simple baseline parameters shown in Table 1. Three distinct clusters of COPD outpatients were identified (e-Fig. 3A, B), primarily separated by differences in sTfR, RDW, RBC count, hemoglobin and hematocrit (Fig. 3A).

Clinical characteristics of the clusters are shown in Table 2. Clusters 1 and 2 had particularly poor survival compared to cluster 3 (*p* < 0.001 and *p* = 0.028, respectively) (Fig. 3B). Cluster 3 had lower mPAP, NT-proBNP and CRP levels than cluster 1 and 2 (*p* < 0.001). Cluster 2 was characterized by particularly poor FEV1, DLCO and pulmonary hemodynamics. Cluster 1, in contrast, showed only mild airway obstruction, mild PH and mild loss of DLCO, but very poor survival. Excluding sTfR1 from the analysis resulted in separation of the cohort into 9 clusters (e-Fig. 3C, D), suggesting that sTfR1 has an important role in the distinction of phenotypes in COPD-PH.

**Table 2:** Cluster characteristics of the outpatient COPD cohort

	Cluster 1	Cluster 2	Cluster 3	*P-value*
*N*	23	15	40	*NA*
Sex (*n*), M:F	13:10	14:1	17:23	*0.030*
Age, year	68 (65–72.5)	65 (57.5–68)	68 (63.5–75)	*0.162*
BMI, kg/m^2^	25.46 (21.98–34.1)	27.34 (23.4–30.7)	25.03 (20.51–28.86)	*0.159*
FEV1, % pred	56.7 (47.7–65.2)	52.7 (43.4–62,7)	60.7 (35.5–76.8)	*0.806*
FEV1/FVC	0.62 (0.55–0.66)	0.49 (0.41–0.63)	0.55 (0.45–0.65)	*0.160*
DLCO, % pred	53.4 (33.1–65.9)	38.2 (25.8–45.7)	45.9 (33.4–67.1)	*0.148*
NT-proBNP, pg/mL	1108 (386–3882)	1516 (740–1887)	225 (103–522)	< *0.001*
Hemoglobin, g/dL	11.8 (10.9–12.8)	16.1 (15.3–17.1)	14.0 (13.1–14.7)	< *0.001*
Hematocrit, %	36.8 (33.9–39.2)	48.7 (45.8–51.1)	41.8 (39.1–42.7)	< *0.001*
RBC, million/mm^3^	4.30 (3.88–4.53)	5.62 (5.01–5.85)	4.57 (4.44–4.72)	< *0.001*
RDW, %	16.2 (15.2–17.7)	15.2 (14.3–16.8)	14.0 (13.3–14.5)	< *0.001*
Bilirubin, mg/dL	0.64 (0.35–1.04)	0.77 (0.65–0.99)	0.56 (0.39–0.75)	*0.051*
Uric acid, mg/dL	7.0 (6.0–8.2)	6.7 (5.4–9.7)	5.6 (4.7–7.1)	*0.014*
CRP, mg/L	8.7 (3.3–18.5)	5.9 (2.1–8.9)	1.7 (1.0–3.2)	< *0.001*
sTfR1, mg/L	3.74 (3.13–4.75)	3.41 (2.42–3.62)	2.23 (1.89–2.53)	< *0.001*
mPAP, mmHg	38 (28.5–50)	41 (36.6–46)	27 (21.8–35)	< *0.001*
PVR, Wood unit	4.49 (4.23–8.21)	7.55 (6.03–9.17)	3.91 (2.62–5.17)	< *0.001*
CI, L/min/m^2^	2.3 (2.0–3.1)	2.3 (1.7–2.7)	2.6 (2.1–2.8)	*0.272*
PAWP, mmHg	11.0 (7.8–14.0)	10.0 (9.0–12.0)	9.0 (7.0–10.8)	*0.050*

Data are presented as n or median with interquartile ranges (IQR, Q1-Q3). Differences between clusters are assessed using the Chi-square and the Kruskal–Wallis tests. *BMI* body mass index, mPAP, mean pulmonary artery pressure, *PVR* pulmonary vascular resistance, *CI* cardiac index, *FEV1* forced expiratory volume in 1 s, *FEV1/FVC* forced expiratory volume in 1 s to forced vital capacity ratio, *DLCO* diffusing capacity of the lung for carbon monoxide, *Hb* hemoglobin, *RBC* red blood cell count, *RDW* red blood cell distribution width, *NT-proBNP* N-terminal pro B-type natriuretic peptide, *CRP* C-reactive protein, *sTfR1* soluble transferrin receptor-1, *PAWP* pulmonary arterial wedge pressure, *NA* not applicable, *pred* predicted

### Transferrin Receptor-1 and Iron Accumulation in Explanted COPD Lungs

**Fig. 4:**
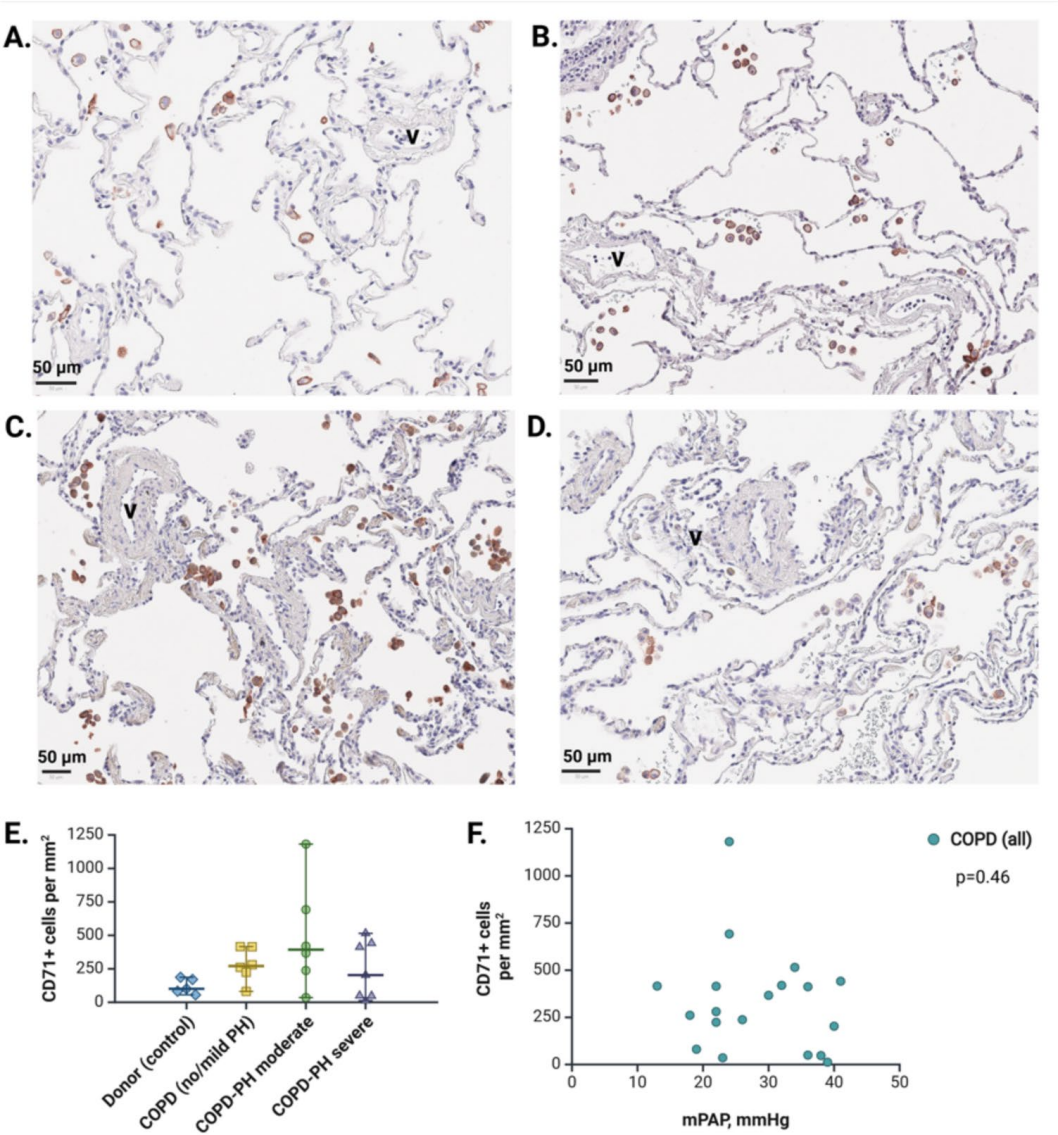
Tissue transferrin receptor-1 in LTX cohort. (**A**) Representative image of IHC staining for CD71 in the lung tissue of healthy donor. Scale: 50 µm. (**B**) Representative image of IHC staining for CD71 in the lung tissue of end-stage COPD patient with no/mild PH. Scale: 50 µm. (**C**) Representative image of IHC staining for CD71 in the lung tissue of end-stage COPD patient with moderate PH. Scale: 50 µm. (**D**) Representative image of IHC staining for CD71 in the lung tissue of end-stage COPD patient with severe PH. Scale: 50 µm. (**E**) Quantification of CD71-positive cells representing transferrin receptor-1 in lung tissues. (**F**) Correlation between the density of CD71-positive cells in the lung and mPAP in COPD. *mPAP* mean pulmonary artery pressure, *V* vessel

We performed immunohistochemical staining of 20 explanted lungs from end-stage COPD patients (Fig. 4A–D) who underwent RHC during their hospitalization before LTX. All COPD subgroups were well-balanced in terms of age and sex, with no significant differences in lung function and iron-associated parameters (e-Table 1). Based on spatial distribution, cell morphology and gene expression signature from a publicly available single-cell RNA sequencing dataset (e-Fig. 4), CD71-positive cells, most likely, corresponded to macrophages, with no subgroup differences (Fig. 4E) or correlation with mPAP (Fig. 4F).

**Fig. 5:**
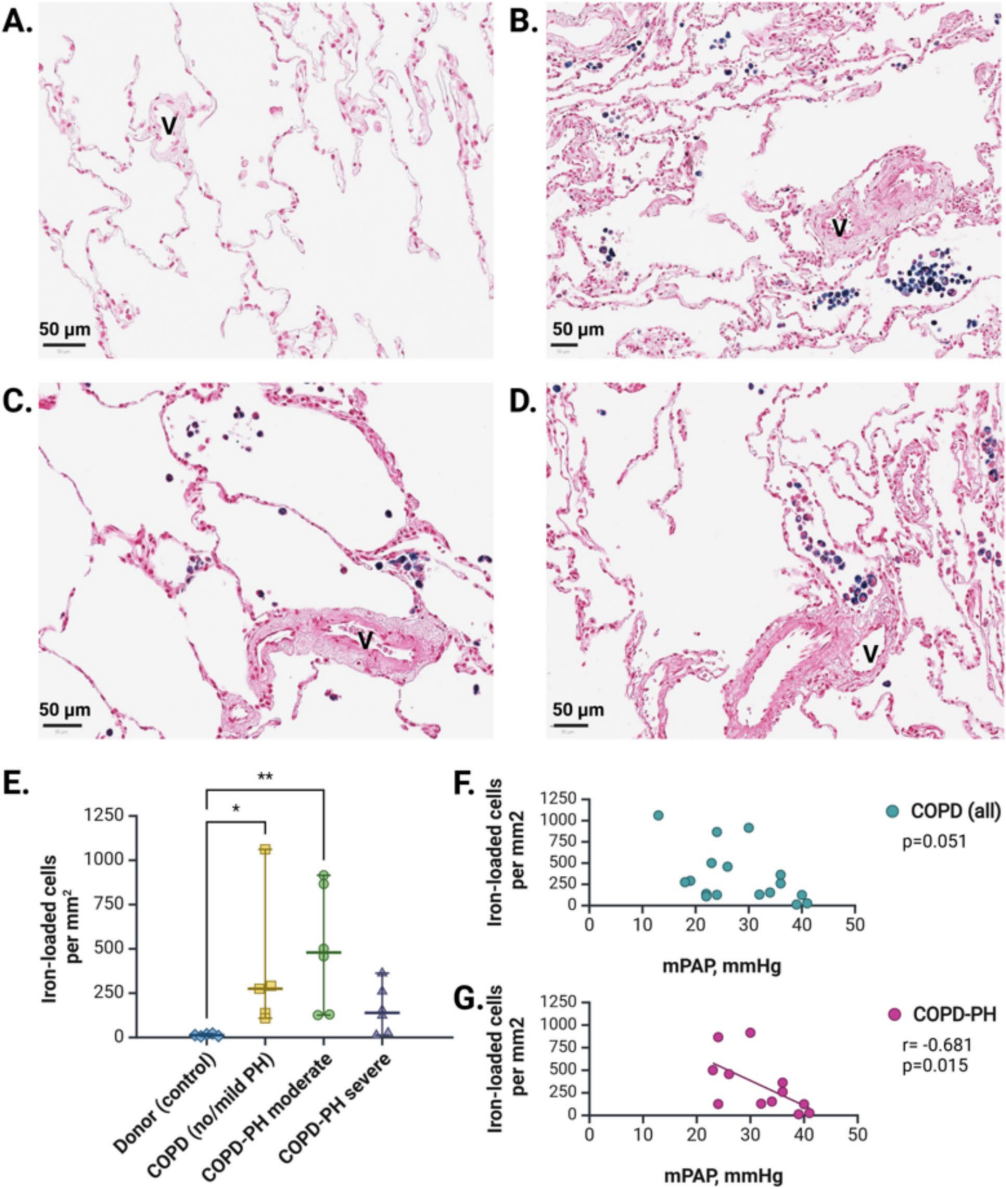
Local iron deposition in LTX cohort. (**A**) Representative image of Perls´ Prussian blue staining for ferric iron in lung tissue from healthy donor. Scale: 50 µm. (**B**) Representative image of Perls´ Prussian blue staining for ferric iron in lung tissue from COPD patient with no/mild PH. Scale: 50 µm. (**C**) Representative image of Perls´ Prussian blue staining for ferric iron in lung tissue from COPD patient with moderate PH. Scale: 50 µm. (**D**) Representative image of Perls´ Prussian blue staining for ferric iron in lung tissue from COPD patient with severe PH. Scale: 50 µm. (**E**) Quantification of ferric iron-loaded cells in COPD subgroups. (**F**) Correlation between mPAP and the number of iron-loaded cells in COPD patients. (**G**) Correlations between mPAP and iron-loaded cells in the COPD-PH group (moderate and severe COPD-PH). *mPAP* mean pulmonary artery pressure; ns, non-significant; *V* vessel. *P < 0.05, **P < *0.01*

Considering sTfR1 as an indicator of iron demand by peripheral tissues [17], we assessed the distribution of ferric iron-positive cells in the LTX lungs (Fig. 5A–D). Again, iron-loaded cells were almost exclusively alveolar macrophages, according to their intraluminal alveolar localization and typical cell morphology. Their density was significantly increased in COPD with no/mild PH (275 cells/mm^2^, IQR: 123–677; *p* = 0.044) and COPD with moderate PH (479 cells/mm^2^, IQR: 129–878; *p* = 0.006) compared to healthy donors (13 cells/mm^2^, IQR: 7–20), but was not different across COPD-PH subgroups (Fig. 5E). In the subgroup of patients with moderate and severe PH, the number of iron-laden cells was negatively correlated with mPAP (*r* = -0.681; *p* = 0.015) (Fig. 5F, G).

## Discussion

Our study demonstrates that in COPD patients from a PH clinic, circulating sTfR1 is associated with PH and poor prognosis which is independent of age and the degree of airflow obstruction. Although airway obstruction and PH are established prognostic factors, the combination of increased sTfR1 with low hemoglobin and increased inflammation markers further characterizes a subgroup of COPD patients with poor prognosis. This suggests that sTfR1 and iron status play an important role as pathologic factors in PH associated with COPD [23, 24].

COPD-PH patients represent a clinical challenge, as they are not rare and require complex management decisions. While even mild PH is linked to increased morbidity and mortality in COPD [25], targeted PAH therapy may only be considered in severe COPD-PH [26]. At the same time, the diagnosis of PH in COPD patients requires invasive RHC [27], as non-invasive markers have not been broadly validated [28]. Identifying reliable biomarkers for severe COPD-PH is therefore crucial for advancing precision medicine [29].

Transferrin receptor-1 is essential for cellular iron uptake, and its cleaved form in the blood, sTfR1, is primarily established as a marker of erythropoietic activity and iron-deficient anemia [17, 30, 31]. However, growing evidence links iron deficiency to more pronounced pulmonary vascular remodeling and more severe PH [32–35], whereas iron overload in COPD lungs may impair hypoxic pulmonary vasoconstriction, causing low ventilation/perfusion areas, severe hypoxemia and less severe PH [36].

In our study, sTfR1 levels were positively associated with mPAP and PVR, and elevated sTfR1 values indicated poor survival, suggesting that sTfR1 may be considered as a biomarker for severe PH in COPD. This is supported by an animal model where *TfR1*^±^ mice exposed to hypoxia showed attenuated pulmonary vascular remodeling and right ventricular systolic pressure [37].

In addition, circulating sTfR1 was correlated with RDW, another factor linked to iron metabolism and PH [23]. Both factors had a significant predictive value for severe PH. However, RDW is a non-specific marker of dysregulated iron metabolism, particularly in the context of inflammatory diseases [38], whereas sTfR1 may remain unaffected by inflammation [39], making it a more reliable marker for PH. Elevated sTfR1 values have also been reported in idiopathic PAH [40], further supporting the involvement of iron deficiency in pulmonary vascular remodeling. Surprisingly, sTfR1 levels were associated with mPAP only in non-anemic COPD outpatients, suggesting that anemia per se is a strong prognostic factor.

Due to the complex associations between sTfR1, pulmonary hemodynamics and other clinical parameters, we performed PCA and unsupervised clustering and identified three distinct groups, mainly driven by sTfR1, RDW, RBC count, hemoglobin and hematocrit. sTfR and RDW loading vectors grouped together, while hemoglobin, hematocrit and RBC vectors were almost orthogonal to this, suggesting an independent role of iron regulating factors and actual red blood cell mass. The survival rate in the three clusters was significantly different, with a better survival in cluster 3. This may be explained by mild airway obstruction and mild PH, as compared to cluster 2. In contrast, cluster 1 was associated with poor survival, despite relatively mild airway obstruction and PH. This cluster was characterized by the lowest hemoglobin and hematocrit levels and the highest CRP and sTfR1 levels of all clusters, suggesting that the combination of iron deficiency, anemia and systemic inflammation might represent a distinct phenotype that requires special attention and may benefit from different therapeutic approaches.

In the explanted COPD lungs, we found that both cellular TfR1 and ferric iron appeared to be predominantly bound to alveolar macrophages, and that iron deposition was inversely correlated with mPAP in COPD-PH patients. This suggests a potential link between local iron deficiency and pulmonary vascular remodeling.

### Limitations

Our COPD cohort was relatively small and enriched with severe PH without severe comorbidities. However our cohort was deeply phenotyped, including RHC, a detailed clinical workup and a long follow up. Validation in a larger independent cohort is warranted. In the explanted COPD lungs, in many cases, reliable PAWP values were not available. This precluded analysis on the role of pulmonary capillary pressure on the number of iron-loaded macrophages.

## Conclusions

In patients with COPD, elevated sTfR1 represents a predictor of severe PH, particularly if combined with decreased hemoglobin levels. COPD-PH patients with signs of systemic inflammation, iron deficiency and anemia may represent a distinct phenotype with poor prognosis, independent of airway obstruction and pulmonary hypertension. This phenotype may benefit from specific therapeutic strategies.

## Supplementary Information

Below is the link to the electronic supplementary material.

Supplementary file1 (DOCX 880 KB)

## Data Availability

All data generated or analyzed during this study are included in this published article and its supplementary files. Further inquiries can be directed to the corresponding author.

## Abbreviations

AUROC: Area Under Receiver Operating Characteristic Curve
BMI: Body Mass Index
COPD: Chronic Obstructive Pulmonary Disease
COPD-PH: Pulmonary Hypertension Associated with COPD
CI: Cardiac Index
CRP: C-Reactive Protein
DLCO: Diffusing Capacity of the Lung for Carbon Monoxide
FEV1: Forced Expiratory Volume in 1 s
FEV1/FVC: Forced Expiratory Volume in 1 s to Forced Vital Capacity Ratio
GOLD: Global Initiative for Chronic Obstructive Lung Disease
HIF-2: Hypoxia-Inducible Factor-2
IHC: Immunohistochemistry
LTX: Lung Transplant Cohort
mPAP: Mean Pulmonary Artery Pressure
NT-proBNP: N-Terminal pro-B-Type Natriuretic Peptide
NA: Not Applicable
PH: Pulmonary Hypertension
PAH: Pulmonary Arterial Hypertension
PCA: Principal Component Analysis
PVR: Pulmonary Vascular Resistance
RBC: Red Blood Cells
RDW: Red Blood Cell Distribution Width
RHC: Right Heart Catheterization
ROC: Receiver Operating Characteristic Curve
sTfR1: Soluble Transferrin Receptor-1
TfR1: Transferrin Receptor-1
pred: Predicted
V: Vessel
WHO: World Health Organization
