# Supplementary material for: Soluble Transferrin Receptor-1 in Pulmonary Hypertension Associated with COPD
Source: Lung. 2025 Jul 14;203(1):79. doi: 10.1007/s00408-025-00833-3 (PMC12259483; doi:10.1007/s00408-025-00833-3)
Supplement: Supplementary file 1 — Supplementary file1 (DOCX 880 KB) [file 408_2025_833_MOESM1_ESM.docx]

**SUPPLEMENTARY MATERIALS**

**e-Figure 1.** Generalized Additive Model of non-linear relationship between sTfR1 and mPAP in COPD


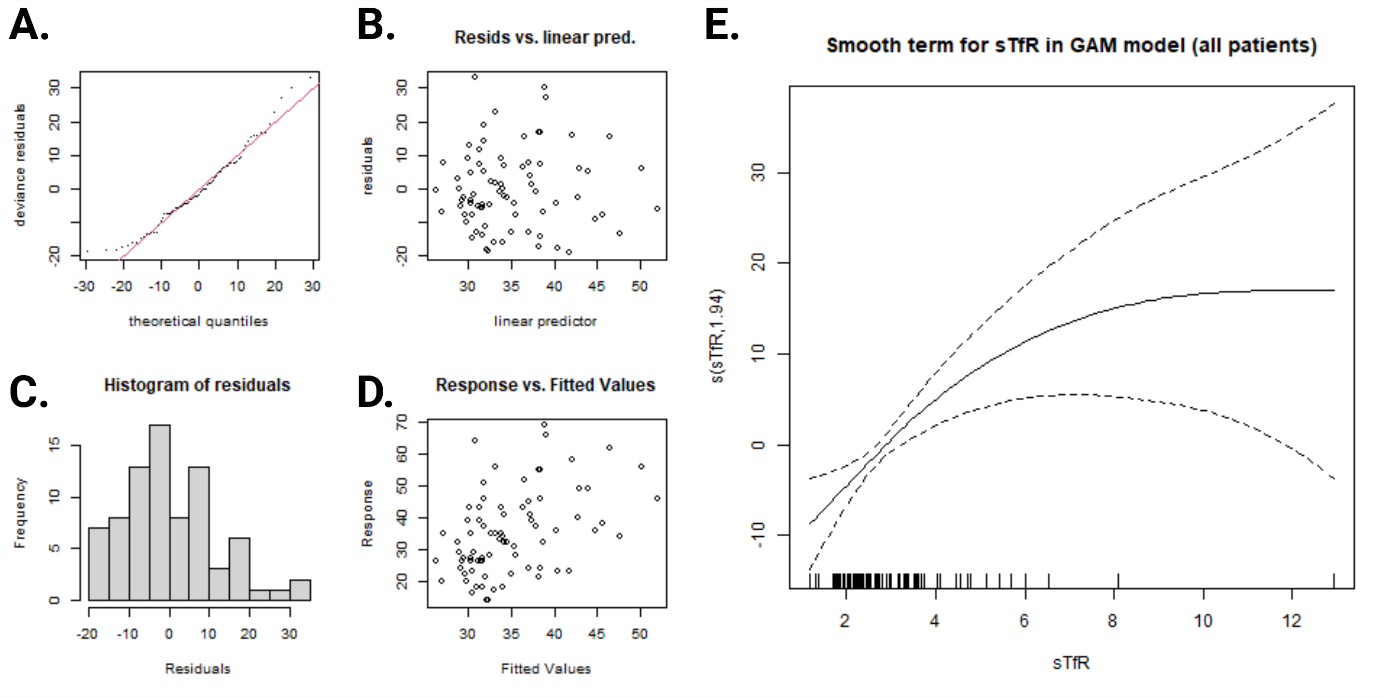


(A) Q-Q plot assessing the normality of residuals. (B) Residuals vs. fitted values plot evaluating homoscedasticity. (C) Histogram of residuals assessing their distribution. (D) Response vs. fitted values plot illustrating the model fit. (E) Smooth term plot for the GAM model showing the relationship between the response variable (mPAP) and the predictor sTfR1. The spline smoother (s(sTfR)) represents the estimated smooth function of GAM with 95% confidence intervals.

**e-Figure 2.** Associations between sTfR1 and anemia, BMI, GOLD stages


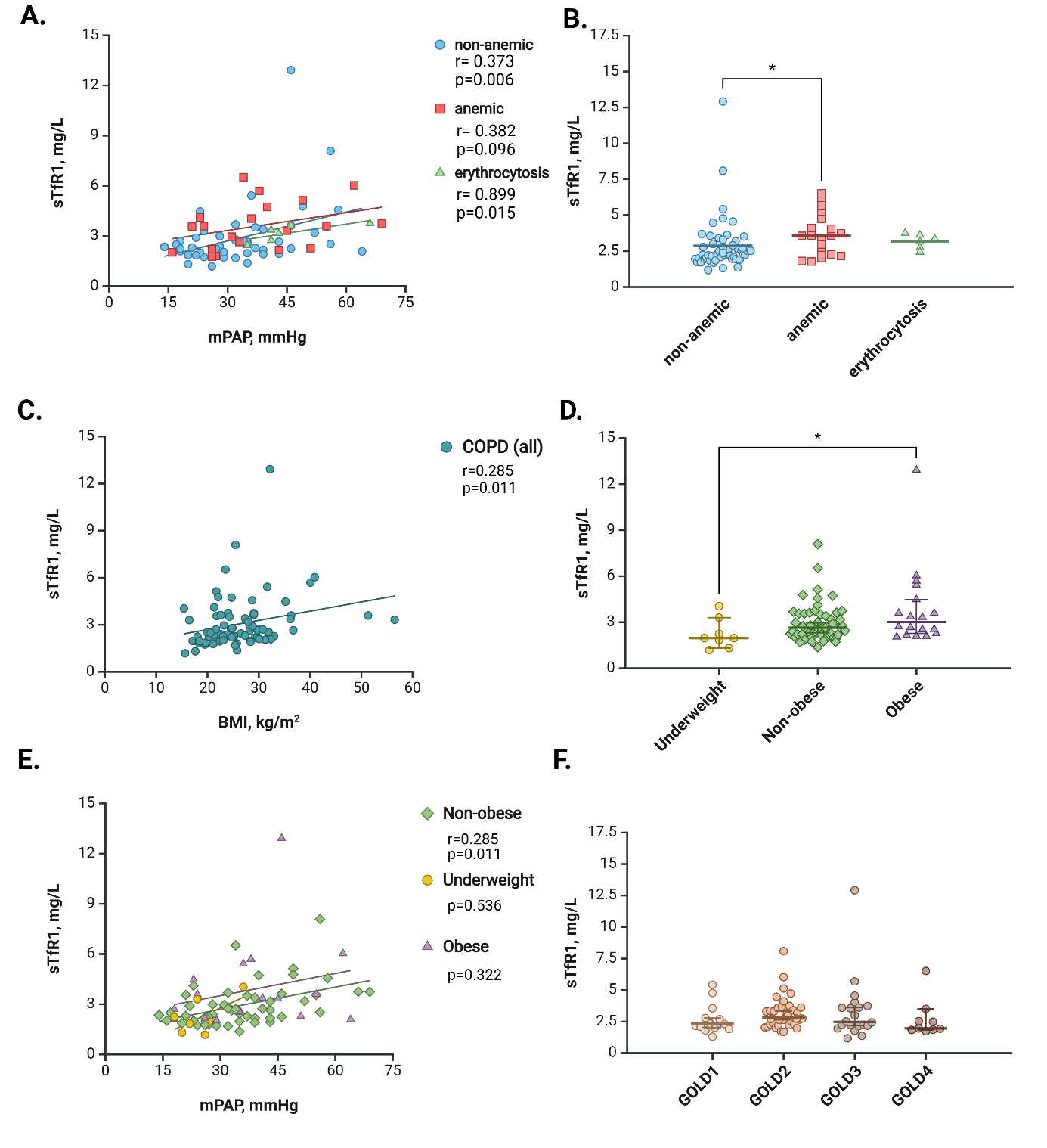


(A) Correlation between sTfR1 levels and mPAP in anemic, non-anemic COPD patients and patients with erythrocytosis. (B) Comparison of sTfR1 levels between anemic, non-anemic COPD patients and patients with erythrocytosis. (C) Correlation between sTfR1 levels and body mass index (BMI). (D) sTfR1 in underweight (BMI<18.5 kg/m^2^, n=8) vs. non-obese (BMI=18.5-29.9 kg/m^2^, n=52) vs. obese COPD patients (BMI>30 kg/m^2^, n=18). (E) Correlation between sTfR1 and mPAP within underweight, non-obese and obese COPD patients. (F) Comparison of sTfR1 levels between COPD patients with different GOLD stages (GOLD1-4). BMI, body mass index; RDW, red blood cell distribution width; GOLD, Global initiative for chronic Obstructive Lung Disease; mPAP, mean pulmonary artery pressure; ns, non-significant. *P < 0.05, **P < 0.01, ***P < 0.001.

**e-Figure 3.** Unsupervised clustering of COPD outpatients


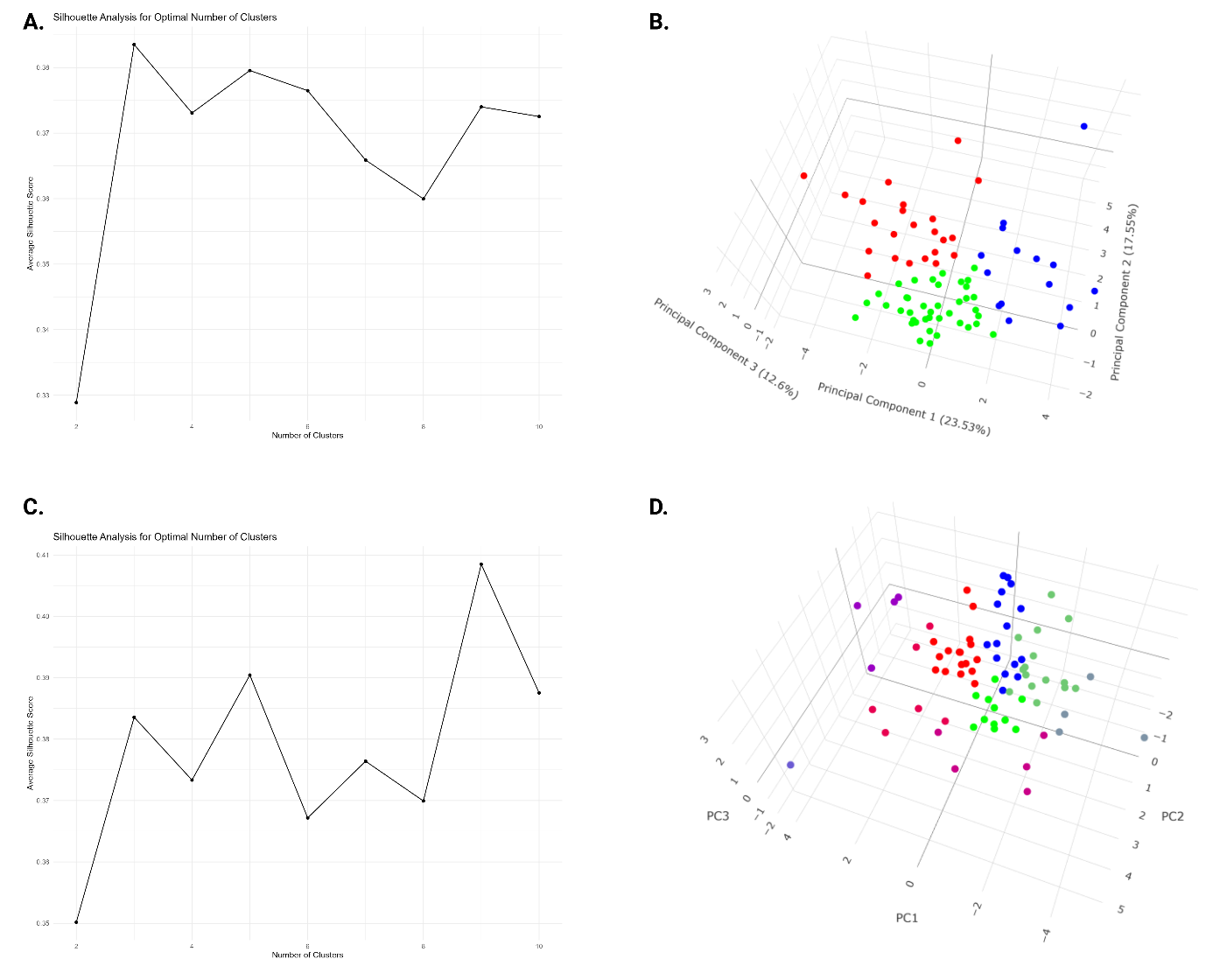


(A) Silhouette plot for the optimal number of clusters based on non-hemodynamic parameters including sTfR1. (B) 3D PCA plot and unsupervised clustering of COPD outpatients based on non-hemodynamic parameters including sTfR1. (C) Silhouette plot for the optimal number of clusters based on non-hemodynamic parameters excluding sTfR1. (D) 3D PCA plot and unsupervised clustering of COPD outpatients based on non-hemodynamic parameters excluding sTfR.

**e-Table 1.** Demographic and clinical characteristics of the LTX cohort (n=20)

|  | **COPD with no**  **or mild PH** | **COPD-PH**  **moderate** | **COPD-PH**  **severe** | **P-value** |
| --- | --- | --- | --- | --- |
| N | 6 | 7 | 7 | NA |
| Sex (n), M:F | 4:2 | 4:3 | 4:3 | NA |
| Age, years | 56.3 (54.2 - 60.6) | 61.8 (54.5 - 64.8) | 56.5 (55.0 - 63.3) | ns |
| mPAP, mmHg | 20.5 (16.8 - 22.0) | 26.5 (23.8 - 30.5) | 38.0 (36.0 - 40.0) | < 0.01 |
| FEV1, % pred. | 17.7 (13.5 - 30.6) | 22.7 (15.8 - 28.4) | 18.9 (16.0 - 19.2) | ns |
| FEV1/FVC | 0.35 (0.29 - 0.41) | 0.34 (0.32 - 0.41) | 0.33 (0.28 - 0.40) | ns |
| GOLD stage (n) | GOLD3 (2),  GOLD4 (4) | GOLD4 (7) | GOLD4 (7) | NA |
| Hb, g/dL | 14.9 (11.7 – 15.6) | 13.4 (10.9 – 14.1) | 13.7 (12.0 – 15.9) | ns |
| RDW, % | 13.8 (13.4 – 15.4) | 13.8 (12.8 – 15.4) | 14.1 (13.3 – 14.6) | ns |
| Serum iron, µg/dL | 63.0 (49.8 – 97.0) | 68.0 (39.5 – 73.3) | 117.0 (67.5 – 136.0) | ns |
| Ferritin, µg/L | 145.5 (93.8 – 244.0) | 135.5 (60.3 – 464.5) | 203.5 (103.8 – 305.5) | ns |
| Transferrin, mg/dL | 1.97 (1.38 – 2.15) | 1.67 (1.33 – 1.99) | 2.06 (1.69 – 2.45) | ns |

Data are presented as n or median with interquartile ranges (IQR, Q1-Q3). Differences between groups were assessed using the Kruskal-Wallis test. Abbreviations: mPAP, mean pulmonary artery pressure; FEV1, forced expiratory volume in 1 sec; FEV1/FVC, forced expiratory volume in 1 sec to forced vital capacity ratio; GOLD, Global Initiative for Chronic Obstructive Lung Disease; Hb, hemoglobin; RDW, red blood cell distribution width; NA, not applicable; ns, non-significant; pred., predicted.

**e-Figure 4.** Expression pattern of *TFRC* and *SIGLEC1* in human healthy lungs


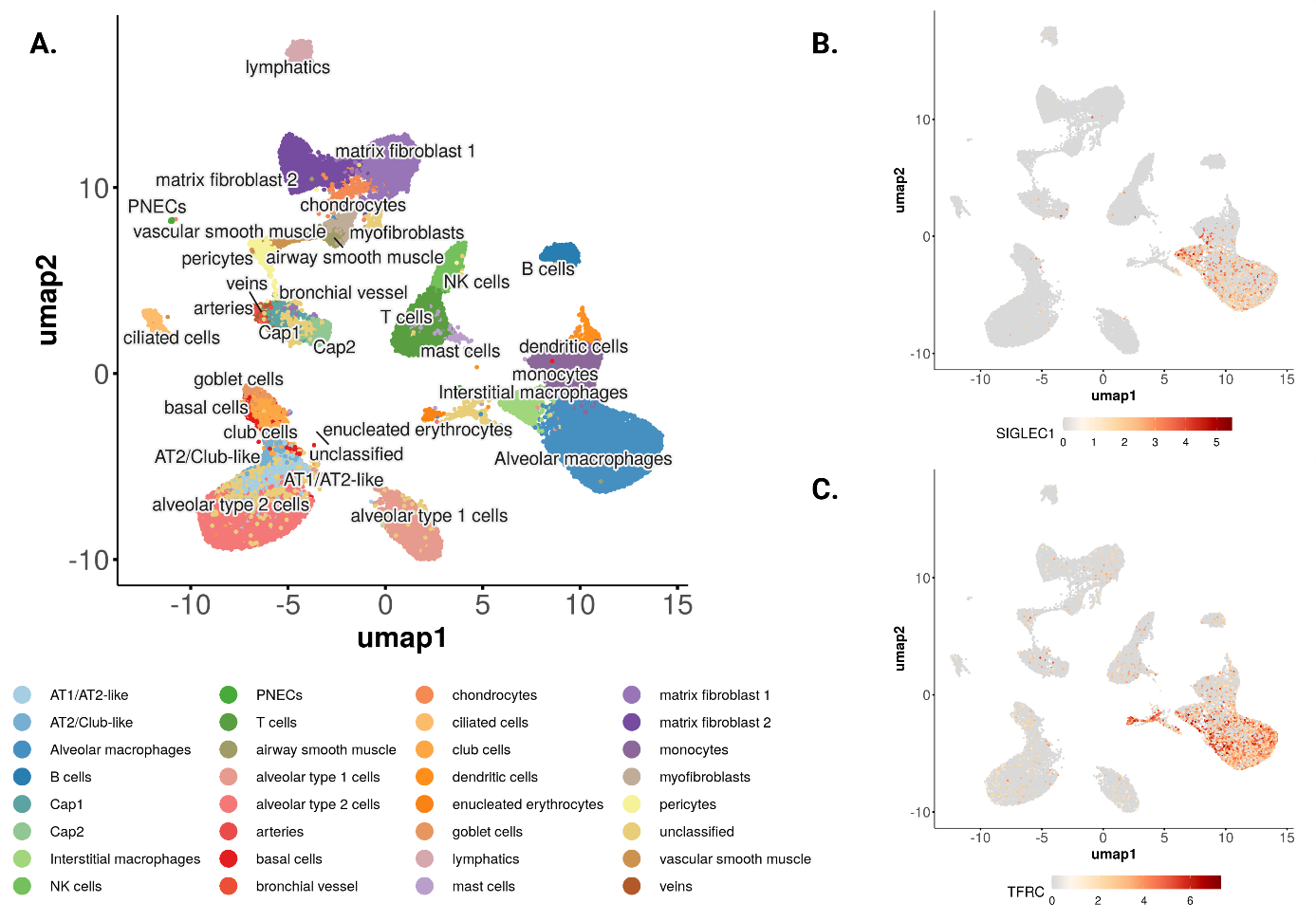


(A) UMAP of different cell types in the LungMap dataset (human data from a broad age healthy donor group). (B) UMAP of the expression levels of lung macrophage marker CD169 (encoded by *SIGLEC1*). (C) UMAP of the expression levels of TfR1/CD71 (encoded by *TFRC*).

The plots have been generated using data from the LungMAP Consortium [1] and downloaded from the ShinyCell LungMAP browser ([ShinyCell LungMAP — Human data from a broad age healthy donor group - Default](https://app.lungmap.net/app/shinycell-lungmap-single-cell-multiomic)), accessed on May 12, 2025. The LungMAP consortium, the Human Tissue Core (U01-HL144861), and the LungMAP Data Coordinating Center (U24-HL148865) are funded by the National Heart, Lung, and Blood Institute.

Reference:

1. Sun X, Perl A-K, Li R, et al (2022) A census of the lung: CellCards from LungMAP. Dev Cell 57:112-145.e2
